# Inflammatory marker dynamics in shift work sleep disorder: a data-driven approach among rotating shift workers

**DOI:** 10.3389/fnins.2026.1764454

**Published:** 2026-03-18

**Authors:** Yiren Bao, Bo Liang, Tianhao Zhan, Guan Shen, Dai Sun, Jialong Lin, Linlin Hu, Rui Wang

**Affiliations:** 1 Department of Massage, The Hangzhou TCM Hospital Affiliated to Zhejiang Chinese Medical University, Hangzhou, Zhejiang, China; 2 Department of Nephrology, The Key Laboratory for the Prevention and Treatment of Chronic Kidney Disease of Chongqing, Chongqing Clinical Research Center of Kidney and Urology Diseases, Xinqiao Hospital, Army Medical University (Third Military Medical University), Chongqing, China; 3 Department of Health Career Development, Hangzhou TCM Hospital Affiliated to Zhejiang Chinese Medical University, Hangzhou, Zhejiang, China; 4 Sleep Medicine Center, Hangzhou TCM Hospital Affiliated to Zhejiang Chinese Medical University, Hangzhou, China

**Keywords:** circadian rhythm, cross-sectional studies, inflammation, shift work schedule, sleep disorders

## Abstract

**Background:**

Shift work sleep disorder (SWSD) negatively affects overall health and quality of life. This cross-sectional study aimed to elucidate the association of SWSD with blood cell counts (BCCs) and related inflammatory indices over a period of 3 years.

**Method:**

This retrospective cross-sectional study involved rotating shift workers. Linear mixed-effects models were applied to examine the associations between group stratification (based on sex and sleep status) and longitudinal trajectories of inflammation indices.

**Results:**

Participants with SWSD (regardless of sex) showed significantly steeper increases in neutrophil levels during the second year (female: *β* = 0.079, *p* = 0.030; male: *β* = 0.076, *p* = 0.032). By the third year, marked changes were observed in monocyte levels, although these were not statistically significant in mixed models. Composite inflammatory indices, including the neutrophil-to-lymphocyte ratio, monocyte-to-lymphocyte ratio, systemic immune-inflammation index, systemic inflammation response index, systemic inflammatory composite index, and neutrophil-to-monocyte ratio, exhibited consistent and significant upward trends in the SWSD group (all *p* < 0.05, adjusted for false discovery rate). Notably, the platelet-to-lymphocyte ratio showed a sex-specific increase only in males with SWSD (*β* = 7.310, *p* = 0.006).

**Conclusion:**

Long-term rotating shift workers with sleep disorders exhibited fluctuating trends in BCCs and related inflammatory indices over time, with neutrophil and monocyte counts showing increases. These patterns suggest that dynamic alterations in BCCs and related inflammatory indices are associated with circadian disruption among rotating shift workers and may have potential relevance for future risk stratification and monitoring.

## 1 Introduction

Shift work constitutes an important organizational model that maintains critical industries such as the healthcare, transportation, and manufacturing sectors (Wright et al., 2013). Researchers estimate that about 20% of workers engage in shift work, with night shifts (11 p.m.–7 a.m. or 12 a.m.–8 a.m.) and rotating shifts (alternating between evening, night, and day shifts) being prevalent (Costa, 2010; Khemakhem et al., 2025). Shift work alters normal sleep patterns and circadian rhythms, resulting in shift work sleep disorder (SWSD). Specifically, this disorder causes difficulties falling and staying asleep, which are often accompanied by excessive daytime sleepiness (Reynolds et al., 2024). Approximately, 20–30% of shift workers face various sleep-related issues, a prevalence substantially higher than that observed in non-shift workers (Reynolds et al., 2024).

Circadian rhythms coordinate not only sleep–wake behavior but also immune homeostasis. Sleep disorders resulting from chronic disruption of circadian rhythm can activate the hypothalamic–pituitary–adrenal (HPA) axis and sympathetic nervous system, thereby placing the body in a state of chronic low-level inflammation. This state is closely associated with several health risks, including metabolic syndrome, cardiovascular disease, cognitive decline, depression, or tumors (Andreadi et al., 2025). Therefore, inflammation is now recognized as a key mechanism contributing to the health risks associated with shift work (Atwater et al., 2021).

Circulating leukocyte subsets also display pronounced circadian rhythmicity. These fluctuations are jointly governed by central and peripheral clocks and are modulated by rhythmic neuroendocrine outputs (e.g., cortisol secretion and sympathetic tone), which regulate leukocyte trafficking and redistribution between blood and tissues (Lange et al., 2022). Such diurnal variation implies that circadian disruption may not only introduce time-related variability in complete blood cell count (BCC)–derived measures, but—when sustained—may also drive longer-term alterations in immune homeostasis (Wang et al., 2022). These changes may be detectable using serial measurements obtained over multiple years (Mok et al., 2024).

Recent studies leveraged accessible BCCs and related composite inflammatory indices; these indices include the neutrophil-to-lymphocyte ratio (NLR), monocyte-to-lymphocyte ratio (MLR), systemic immune-inflammation index (SII), and systemic inflammation response index (SIRI) (Liu et al., 2024; Zhang et al., 2025). For example, Atwater et al. (2021) compared BCCs and plasma inflammatory markers, including white blood cell (WBC) count, monocyte (MC) count, and C-reactive protein, as well as the immune response to *in vitro* stimulation with endotoxins between day-shift and rotating-shift workers in a cohort of 104 healthcare workers (Liu et al., 2024). They found that shift work increases the risk of chronic diseases by inducing low-grade systemic inflammation and disrupting immune responses. Unlike prior studies relying on single time-point measurements, we leveraged historical serial BCCs and composite inflammatory indices recorded over the prior 3 years, enabling us to characterize time-related patterns associated with SWSD (Atwater et al., 2021; Akbar and Shi, 2024; Li J. et al., 2024).

Systematic studies investigating the long-term relationship between SWSD and inflammatory indices remain limited, particularly with respect to how this relationship changes over time. Therefore, using annual BCCs records in a retrospective design, we compared time-related patterns in BCC components and composite inflammatory indices between shift workers with and without SWSD.

## 2 Materials and methods

### 2.1 Study design

We conducted a series of informational lectures on sleep health in the workplaces of participants between 2022 and 2024 as part of patient recruitment process for another cohort study which investigated the effects of acupressure on circadian rhythm sleep–wake disorders. During the lectures, participants completed a structured onsite questionnaire survey. Based on the questionnaire data, we identified respondents who began shift work during 2020–2022, 2021–2023, or 2022–2024, completed three consecutive shifts and included them in this retrospective cross-sectional analysis.

The study protocol was approved by the Ethics and Human Participants Committee of Hangzhou TCM Hospital Affiliated to Zhejiang Chinese Medical University (Approval No. 2024KLL213). The requirement for informed consent was waived because of the retrospective nature of the study. This article was prepared in accordance with the Strengthening the Reporting of Observational Studies in Epidemiology (STROBE) Statement checklist.

### 2.2 Participants

This retrospective cross-sectional study enrolled newly employed rotating shift workers aged 18–40 years from workplaces that participated in a series of sleep health lectures in 2022, 2023, and 2024. Individuals with no history of any sleep disorder prior to employment and no previous systematic treatment for circadian rhythm sleep–wake disorders were eligible for inclusion. Participants were required to have completed at least three consecutive years of shift work, with available health examination records covering both the pre-employment baseline and the three-year follow-up period. To minimize the influence of acute conditions on inflammatory indices, individuals with documented acute infections or acute severe illnesses at the time of routine health examinations were excluded.

**Figure 1:**
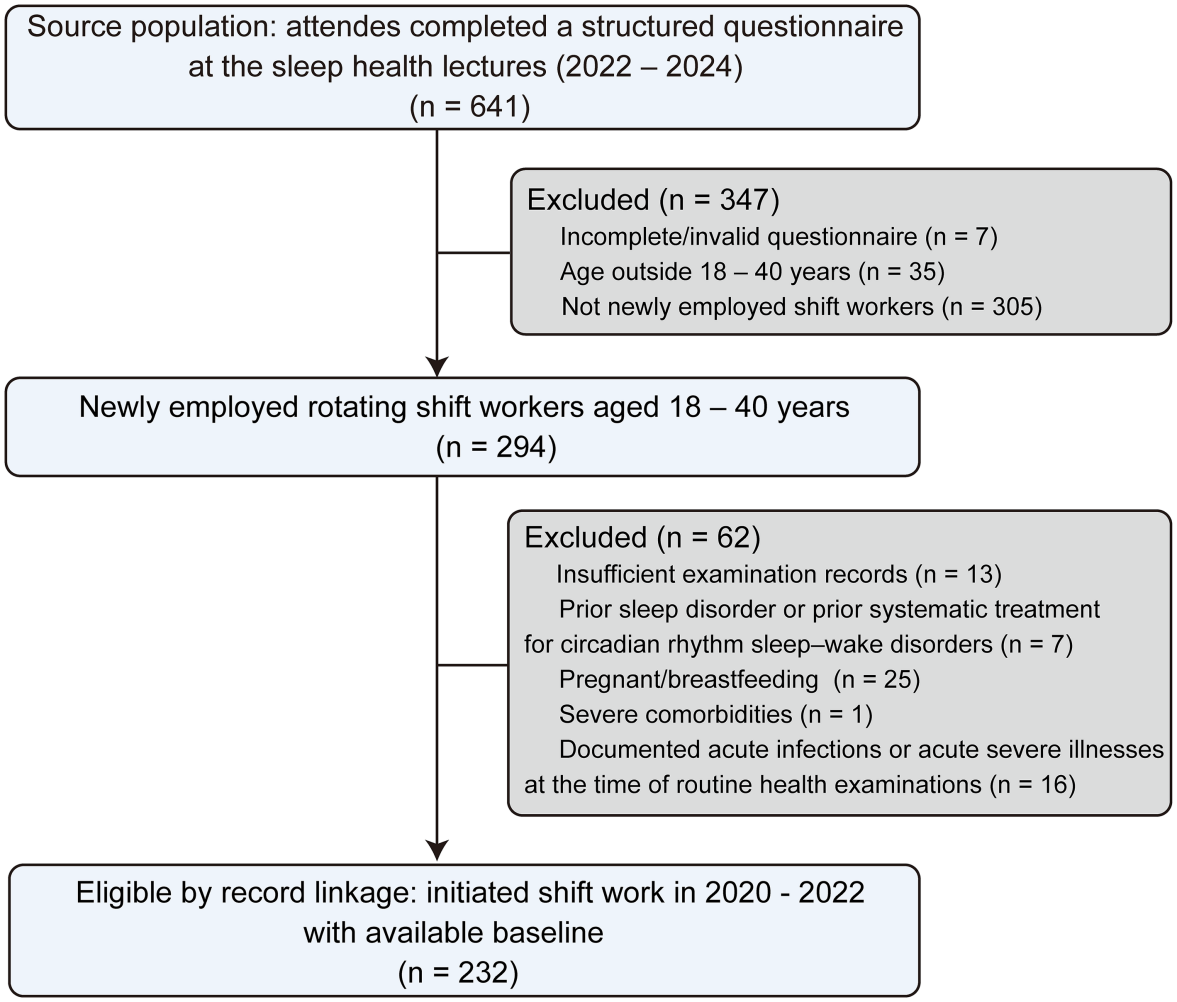
Study flowchart.

Individuals were excluded if they were pregnant or breastfeeding during the study period; had severe comorbidities, including cardiovascular, metabolic, or neoplastic diseases; had not undergone routine health examinations at the company-designated medical institution; provided incomplete questionnaire responses; showed blood test reference ranges inconsistent with general population norms; or had any physician-diagnosed sleep disorder other than shift work disorder. The latter included organic sleep disorders (e.g., obstructive sleep apnea, restless legs syndrome/periodic limb movement disorder, narcolepsy/hypersomnia, and parasomnias) as well as primary insomnia, or current use of continuous positive airway pressure or noninvasive ventilation (American Psychiatric Association, 2013). A total of 232 participants met the eligibility criteria and were included in the final analysis. Participant selection is summarized in Figure 1.

### 2.3 Definition of SWSD

The diagnosis of SWSD, as defined by the International Classification of Sleep Disorders, is based on the following criteria: (1) a complaint of insomnia or excessive sleepiness temporally associated with a recurring work schedule that overlaps the usual time for sleep; (2) symptoms must be associated with the shift work schedule for a duration of ≥ 1 month; (3) circadian and sleep-time misalignment as demonstrated by sleep log or actigraphic monitoring (with sleep diaries) for ≥7 days; (4) this type of sleep disturbance is not explainable by another sleep disorder, a medical or neurological disorder, mental disorder, medication use, or a substance use disorder (American Psychiatric Association, 2013).

### 2.4 Questionnaire

The questionnaire comprised four sections, namely demographic characteristics, general lifestyle and shift work patterns over the past 3 years, the Pittsburgh sleep quality index (PSQI), and annual health examination data collected at baseline and during the three-year follow-up. Given the retrospective design of this study, some variables were classified using simplified categories.

### 2.5 Basic demographic information

Demographic information collected at baseline included age at employment, sex, occupation, and education level. Based on occupation, participants were divided into two groups: factory workers and healthcare workers. Based on education level, they were grouped into two categories: high education level (university degree or above) and mid-level or below (below university).

### 2.6 Lifestyle and shift work

Lifestyle factors collected through the questionnaire included alcohol consumption, smoking status, caffeine intake, and exercise habits. Alcohol consumption and smoking status were separately categorized as non-drinking/non-smoking, infrequent (less than daily) or frequent (daily or almost daily) (Ness-Jensen et al., 2019; Adams et al., 2022). Caffeine intake was assessed based on habitual consumption of caffeine-containing foods and beverages, including coffee, tea, cola, other caffeinated soft drinks, and chocolate, during shift work. Based on the frequency of caffeine intake, participants were categorized as rare, infrequent, or habitual consumers. Exercise habits were classified based on total weekly exercise time. Participants engaging in ≥ 1 h of exercise per week were considered physically active, whereas those engaging in < 1 h per week were considered inactive (Gustad et al., 2014).

Shift work patterns, including non-standard shift work schedules and frequencies, were recorded. Shift schedules were categorized into three types: two-shift (day-evening), three-shift (day–evening-night), or irregular shift schedules. Night-shift frequency was classified as infrequent (< 5-night shifts/month) or frequent (≥ 5-night shifts/month).

### 2.7 Physical and BBCs examinations

Body mass index (BMI) was calculated using the standard formula: weight (kg) divided by height squared (m^2^), based on measurements obtained during routine annual health examinations (Xu et al., 2022). Blood samples were obtained from all participants after an overnight fast in the early morning during the standardized health examination schedule: WBC, neutrophil (NEU), lymphocyte (LC), MC, and platelet (PLT) counts. These values were expressed as 10^3^ cells/μL. Reference ranges for these indices, expressed as 10^9^ cells per liter (×10^9^/L) were as follows: WBC, 3.5–9.5; NEU, 1.8–6.3; LC, 1.1–3.2; MC, 0.1–0.6; and PLT, 125–350.

### 2.8 Assessment of inflammation

Inflammatory indicators were derived from complete blood counts. These indicators included SII, SIRI, systemic inflammatory composite index (AISI), MLR, NLR, neutrophil-to-monocyte ratio (NMLR), and platelet-to-lymphocyte ratio (PLR), as determined using the following formulas (Elangovan et al., 2024; Huang et al., 2025):


SII=(NEU×PLR)/LC

SIRI=(NEU×MC)/LC

AISI=(NEU×MC×PLT)/LC

MLR=MC/LC

NLR=NEU/LC

NMLR=(NEU+MC)/LC

PLR=PLT/LC

### 2.9 Statistical analyses

All statistical analyses were performed using R software (version 4.4.1). Continuous variables were summarized as mean (standard deviation) for normally distributed data or median (interquartile range) for non-normally distributed data. Categorical variables were expressed as frequencies and percentages. Group comparisons were performed using Welch’s *t*-test or ANOVA for normally distributed continuous variables, and the Wilcoxon rank-sum test or Kruskal-Wallis test for non-normally distributed variables. For comparisons of categorical variables between groups, Fisher’s exact test was used when the expected frequencies were < 5; otherwise, the chi-squared test was used. A *p*-value < 0.05 was considered statistically significant.

Participants were divided into groups based on sex and sleep status: males and females, with or without SWSD. Distributions of BCCs and related inflammatory indices were visualized across specific time points within these groups. For each inflammatory marker, a separate linear mixed-effects model was fitted to examine longitudinal changes over time. Repeated measurements were modeled with random intercepts for subject ID to account for correlations between measurements within individuals. Fixed effects included interaction terms between the group variable (comprising combinations of sex and sleep status) and time, allowing for group-specific time trends.

Estimated marginal means and pairwise comparisons were performed using the emmeans package, with adjustment for multiple comparisons using the false discovery rate (FDR). Degrees of freedom were estimated using the Satterthwaite approximation. Model outcomes were visualized using coefficient plots with 95% confidence intervals and interaction plots displaying predicted time trends across groups, incorporating individual-level data.

## 3 Results

### 3.1 Population characteristics and inflammatory indices

Table 1 presents the demographic characteristics of the participants. A significant difference in education level was observed (*p* = 0.008), with the female group having a higher proportion of individuals with higher education (30.3–52.8%) compared with the male group (25.0–25.9%). Occupational distribution also differed significantly (*p* = 0.002), with a higher percentage of healthcare workers in the female group (31.5–56.6%) than in the male group (25.0–25.9%). Smoking and alcohol consumption were more prevalent in the male group (both *p* < 0.001) than in the female group. The proportion of frequent smokers ranged from 46.9–50.0% among males and from 5.7–10.1% among females. The proportion of frequent alcohol consumers was also significantly higher in the male group (31.3–39.7%) than in the female group (5.7–9.0%). Physical activity habits differed significantly between groups (*p* = 0.002), with a higher proportion of active exercisers in the male group (34.4–41.4%) than in the female group (16.9–17.0%).

**Table 1:** Baseline characteristics of the four groups included in the present study.

Characteristics	Groups				*p*-value
	Female with SWSD *n* = 53	Female without SWSD *n* = 89	Male with SWSD *n* = 32	Male without SWSD *n* = 58	
Age, Median (Q1, Q3)	23.00 (22.00, 26.00)	23.00 (22.00, 25.00)	26.00 (22.00, 27.00)	25.00 (21.00, 27.00)	0.096^a^
Education, *n* (%)					
High level	28 (52.8%)	27 (30.3%)	8 (25.0%)	15 (25.9%)	0.008^b^
Mid-level or below	25 (47.2%)	62 (69.7%)	24 (75.0%)	43 (74.1%)	
Occupation, *n* (%)					
Factory employees	23 (43.4%)	61 (68.5%)	24 (75.0%)	43 (74.1%)	0.002^b^
Healthcare workers	30 (56.6%)	28 (31.5%)	8 (25.0%)	15 (25.9%)	
Shift schedule, *n* (%)					
Irregular	3 (5.7%)	8 (9.0%)	2 (6.3%)	7 (12.1%)	0.104^c^
Three shifts	34 (64.2%)	62 (69.7%)	15 (46.9%)	30 (51.7%)	
Two shifts	16 (30.2%)	19 (21.3%)	15 (46.9%)	21 (36.2%)	
Frequency of night shift, *n* (%)					
Frequent	35 (66.0%)	63 (70.8%)	16 (50.0%)	34 (58.6%)	0.151^b^
Infrequent	18 (34.0%)	26 (29.2%)	16 (50.0%)	24 (41.4%)	
Alcohol, *n* (%)					
Frequent drinking	3 (5.7%)	8 (9.0%)	10 (31.3%)	23 (39.7%)	< 0.001^b^
Non-drinking or infrequent	50 (94.3%)	81 (91.0%)	22 (68.8%)	35 (60.3%)	
Smoking, *n* (%)					
Frequent smoking	3 (5.7%)	9 (10.1%)	15 (46.9%)	29 (50.0%)	< 0.001^b^
Non-smoking or infrequent	50 (94.3%)	80 (89.9%)	17 (53.1%)	29 (50.0%)	
Caffeine, *n* (%)					
Habitual	22 (41.5%)	45 (50.6%)	19 (59.4%)	30 (51.7%)	0.435^b^
Rarely or infrequent	31 (58.5%)	44 (49.4%)	13 (40.6%)	28 (48.3%)	
Exercise, *n* (%)					
Active	9 (17.0%)	15 (16.9%)	11 (34.4%)	24 (41.4%)	0.002^b^
Inactive	44 (83.0%)	74 (83.1%)	21 (65.6%)	34 (58.6%)	

Participants were grouped by sex and SWSD status into four categories: females with SWSD (*n* = 53), females without SWSD (*n* = 89), males with SWSD (*n* = 32), and males without SWSD (*n* = 58). SWSD, shift work sleep disorder.Q1 = first quartile (25th percentile); Q3 = third quartile (75th percentile).

a Kruskal-Wallis rank sum test.

b Pearson’s chi-squared test.

c Fisher’s exact test.

**Figure 2:**
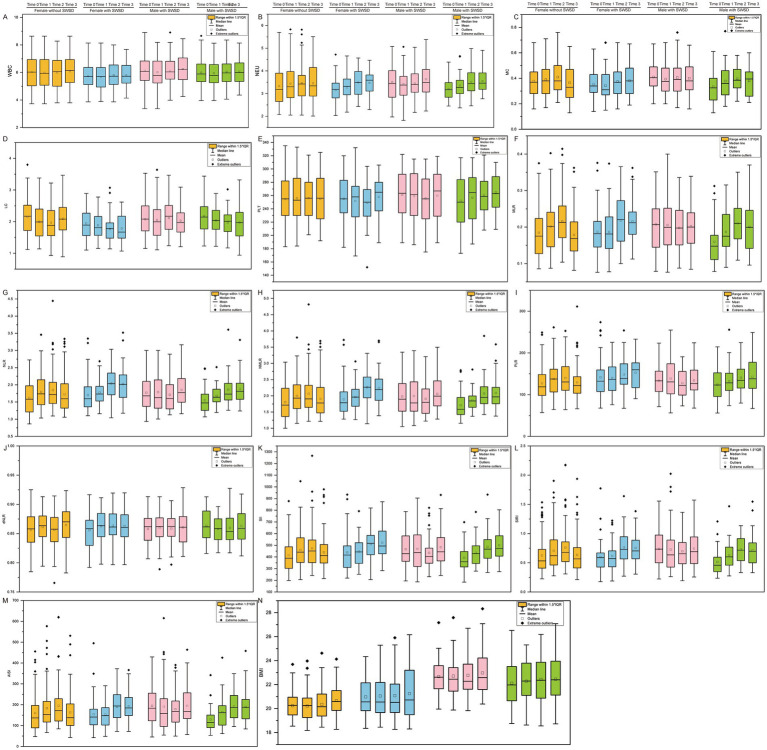
Participant classification based on blood cell counts (BCCs), related inflammatory indices, and body mass index (BMI) from baseline to year 3, stratified by sex and shift work sleep disorder (SWSD) status. **(A)** WBC, **(B)** NEU, **(C)** MC, **(D)** LC, **(E)** PLT, **(F)** MLR, **(G)** NLR, **(H)** NMLR, **(I)** PLR, **(J)** dNLR, **(K)** SII, **(L)** SIRI, **(M)** AISI, and **(N)** BMI. Time 0 indicates baseline. Boxplots display the median (central line), mean (square marker), interquartile range (box), and whiskers extending to 1.5×IQR; points indicate outliers/extreme values. WBC, white blood cell count; NEU, neutrophils; MC, monocytes; LC, lymphocytes; PLT, platelets; BMI, body mass index; MLR, monocyte-to-lymphocyte ratio; NLR, neutrophil-to-lymphocyte ratio; NMLR, neutrophil–monocyte–lymphocyte ratio; PLR, platelet-to-lymphocyte ratio; dNLR, derived neutrophil-to-lymphocyte ratio; SII, systemic immune-inflammation index; SIRI, systemic inflammation response index; AISI, aggregate index of systemic inflammation.

Figure 2 illustrates the changes in BBCs and related inflammatory indices within the first 3 years of employment across different groups. Notably, changes in WBC count (Figure 2A), PLT count (Figure 2E), and dNLR (Figure 2J) were not significant, and no clear temporal fluctuations were observed. In the group with SWSD, both NEU (Figure 2B) and MC (Figure 2C) exhibited a gradual increase over time, a pattern consistent for both male and female participants. Specifically, NEU significantly increased in the second year, whereas MC significantly increased in the third year. The LC (Figure 2D) also showed a marked increase in the third year. However, large individual variations were observed, suggesting substantia variability in the inflammatory response. For several inflammatory indices, such as MLR (Figure 2F), NLR (Figure 2G), NMLR (Figure 2H), PLR (Figure 2I), SII (Figure 2K), SIRI (Figure 2L), and AISI (Figure 2M), the median values in the SWSD group showed a consistent upward trend, with increasing variability over time. This trend was consistently observed across both sexes, suggesting that SWSD may be closely linked to the sustained elevation of systemic inflammation. In addition, no significant changes in BMI (Figure 2N) were observed between groups with and without SWSD over time.

### 3.2 Longitudinal changes across groups

**Figure 3:**
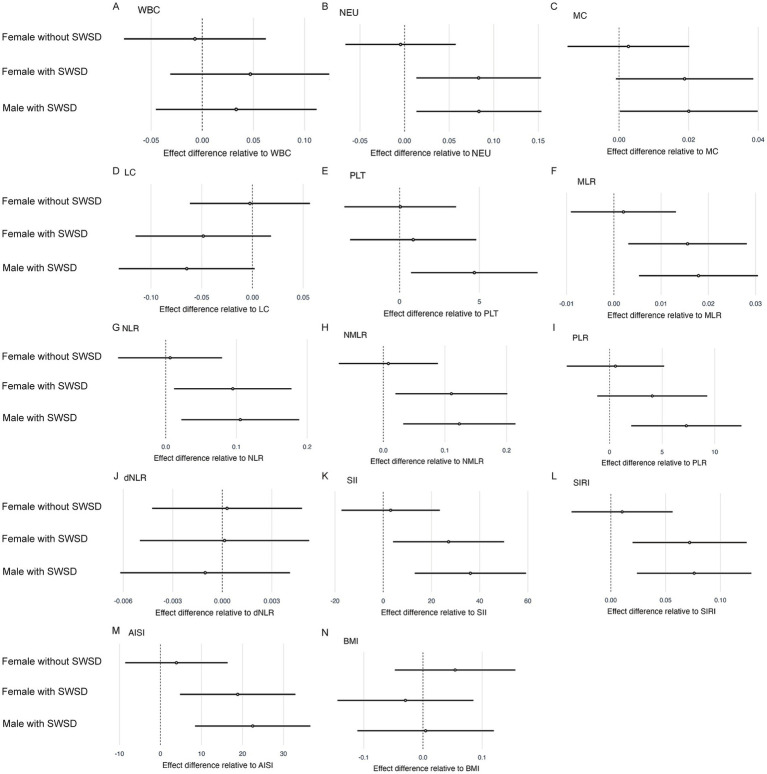
Forest plots showing the effect differences between participant groups. Circles represent coefficients (see Table 2), with horizontal dark lines indicating 95% confidence intervals. **(A)** WBC, **(B)** NEU, **(C)** MC, **(D)** LC, **(E)** PLT, **(F)** MLR, **(G)** NLR, **(H)** NMLR, **(I)** PLR, **(J)** dNLR, **(K)** SII, **(L)** SIRI, **(M)** AISI, and **(N)** BMI.

**Figure 4:**
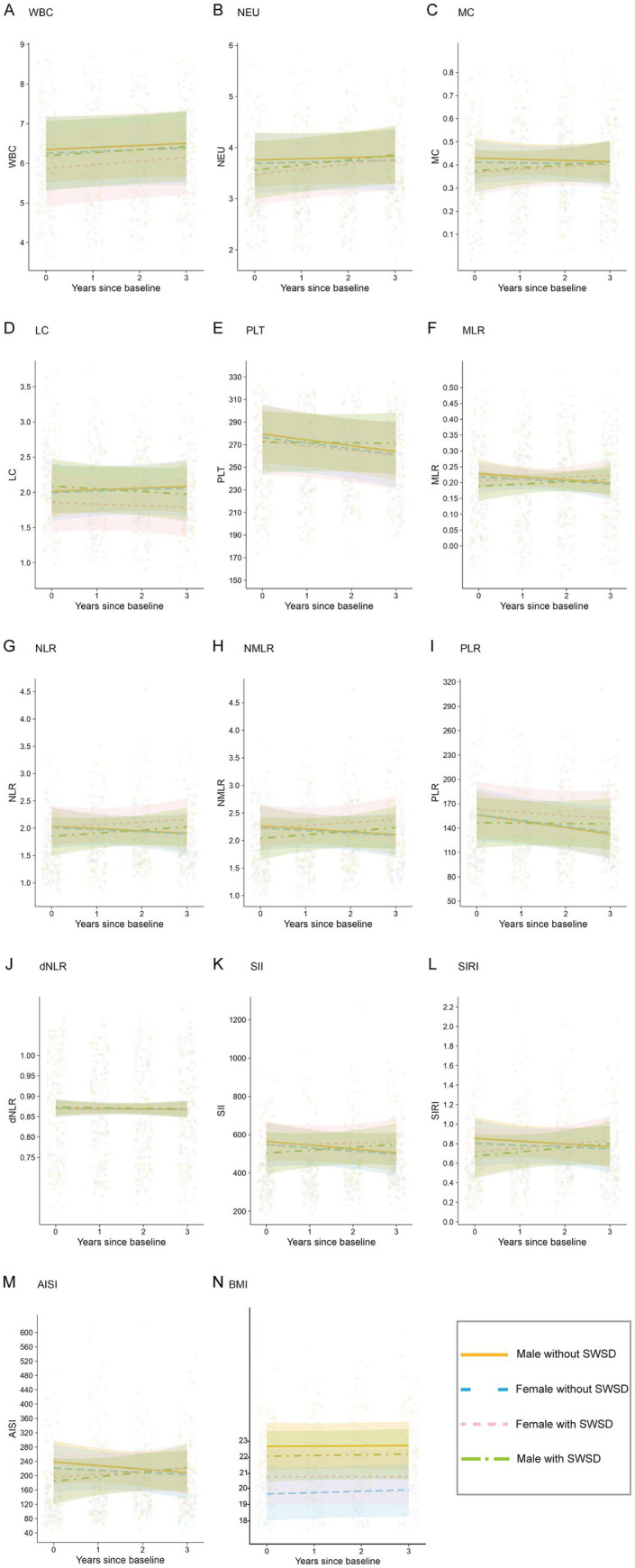
Cluster membership for all blood cell counts, related inflammatory indices, and body mass index over three years. Intercepts and slopes of the four clusters were obtained from linear mixed-effects models. **(A)** WBC, **(B)** NEU, **(C)** MC, **(D)** LC, **(E)** PLT, **(F)** MLR, **(G)** NLR, **(H)** NMLR, **(I)** PLR, **(J)** dNLR, **(K)** SII, **(L)** SIRI, **(M)** AISI, and **(N)** BMI.

Table 2 summarizes the results of the linear mixed-effects models examining the associations between group membership and longitudinal changes in BMI, BCCs and related inflammatory indices. Figure 3 displays forest plots of effect differences between participants, and Figure 4 depicts cluster membership alongside longitudinal changes in inflammatory indices and BMI.

**Table 2:** Summary of linear mixed-effects models.

	**NEU**				**PLT**				**WBC**				**LC**				**MC**			
Predictors	β	95% CI	SE	p-value	β	95% CI	SE	p-value	β	95% CI	SE	p-value	β	95% CI	SE	p-value	β	95% CI	SE	p-value
time: group female without SWSD	−0.005	−0.066 −0.058	0.032	0.888	0.042	-3.456 - 3.540	1.785	0.981	-0.007	-0.077 - 0.062	0.036	0.84	-0.003	-0.062 - 0.057	0.03	0.934	0.003	-0.015 - 0.020	0.009	0.764
time: group female with SWSD	0.079	0.009 −0.149	0.036	0.027	0.895	-3.047 - 4.837	2.011	0.657	0.043	-0.035 - 0.121	0.04	0.283	-0.047	-0.114 - 0.020	0.034	0.168	0.018	-0.002 -0.037	0.010	0.084
time: group male with SWSD	0.076	0.007 −0.146	0.036	0.032	4.752	0.811 - 8.693	2.011	0.018	0.027	-0.052 - 0.105	0.04	0.508	-0.062	-0.129 - 0.004	0.034	0.068	0.018	-0.002 - 0.038	0.010	0.075
time: Education	0.008	−0.101 −0.117	0.056	0.885	3.776	-2.381 - 9.933	3.141	0.23	-0.005	-0.127 - 0.117	0.062	0.937	-0.026	-0.130 - 0.078	0.053	0.628	0.012	-0.019 - 0.043	0.016	0.453
time: Occupation	−0.004	−0.114 −0.107	0.056	0.95	1.774	-4.442 - 7.989	3.171	0.576	-0.007	-0.131 - 0.116	0.063	0.908	-0.003	-0.108 - 0.103	0.054	0.962	0.002	-0.029 - 0.033	0.016	0.891
time: Alcohol	0.024	−0.033 −0.081	0.029	0.414	0.142	-3.098 - 3.382	1.653	0.932	0.053	-0.011 - 0.118	0.033	0.105	0.037	-0.018 - 0.092	0.028	0.188	-0.004	-0.020 - 0.013	0.008	0.652
time: Smoking	0.002	−0.055 −0.059	0.029	0.938	1.122	-2.083 - 4.327	1.635	0.493	-0.041	-0.104 - 0.023	0.033	0.213	-0.045	-0.099 - 0.009	0.028	0.105	0.003	-0.013 - 0.019	0.008	0.742
time: Exercise	−0.008	−0.058 −0.043	0.026	0.762	0.950	-1.895 - 3.795	1.452	0.513	-0.035	-0.091 - 0.022	0.029	0.226	-0.017	-0.065 - 0.031	0.025	0.484	-0.009	-0.023 - 0.005	0.007	0.220

	**dNLR**				**SII**				**SIRI**				**AISI**				**MLR**			
Predictors	β	95% CI	SE	p-value	β	95% CI	SE	p-value	β	95% CI	SE	p-value	β	95% CI	SE	p-value	β	95% CI	SE	p-value
time: group female without SWSD	< 0.001	-0.004 -0.005	0.002	0.902	3.091	-17.302 - 23.483	10.404	0.767	0.010	-0.036 - 0.057	0.024	0.662	3.881	-8.591 - 16.353	6.363	0.542	0.002	-0.009 - 0.013	0.006	0.721
time: group female with SWSD	< 0.001	-0.005 - 0.005	0.003	0.901	26.553	3.572 - 49.534	11.725	0.024	0.069	0.017 - 0.121	0.027	0.010	18.824	4.769 - 32.879	7.171	0.009	0.015	0.002 - 0.028	0.006	0.019
time: group male with SWSD	< 0.001	-0.006 -0.004	0.003	0.781	35.255	12.281 - 58.230	11.722	0.003	0.072	0.019 - 0.124	0.027	0.007	22.481	8.429 - 36.532	7.169	0.002	0.017	0.004 -0.030	0.006	0.008
time: Education	-0.001	-0.009 - 0.007	0.004	0.796	17.291	-18.601 - 53.182	18.312	0.345	0.042	-0.039 - 0.124	0.042	0.307	12.748	-9.203 - 34.699	11.200	0.255	0.011	-0.009 - 0.030	0.010	0.290
time: Occupation	0.001	-0.007 -0.009	0.004	0.877	10.302	-25.931 - 46.535	18.486	0.578	0.017	-0.065 - 0.099	0.042	0.681	4.743	-17.417 - 26.903	11.306	0.675	0.005	-0.015 -0.025	0.010	0.610
time: Alcohol	0.003	-0.002 - 0.007	0.002	0.223	-2.170	-21.057 - 16.717	9.636	0.822	-0.012	-0.055 - 0.031	0.022	0.582	-3.578	-15.129 - 7.974	5.894	0.544	-0.005	-0.015 -0.006	0.005	0.375
time: Smoking	-0.001	-0.005 -0.003	0.002	0.633	10.275	-8.409 - 28.958	9.533	0.282	0.017	-0.025 - 0.060	0.022	0.424	4.534	-6.893 - 15.961	5.830	0.437	0.006	-0.005 - 0.016	0.005	0.280
time: Exercise	0.002	-0.002 - 0.005	0.002	0.352	3.964	-12.622 - 20.550	8.462	0.640	-0.015	-0.052 - 0.023	0.019	0.437	-3.775	-13.918 -6.369	5.175	0.466	-0.003	-0.012 - 0.006	0.005	0.531

	**NLR**				**NMLR**				**PLR**				**BMI**				\			
Predictors	β	95% CI	SE	p-value	β	95% CI	SE	p-value	β	95% CI	SE	p-value	β	95% CI	SE	p-value	\	\	\	\
time: group female without SWSD	0.006	-0.067 -0.080	0.038	0.869	0.008	-0.072 - 0.089	0.041	0.841	0.562	-4.065 - 5.190	2.361	0.812	0.054	-0.048 - 0.157	0.052	0.300	\	\	\	\
time: group female with SWSD	0.092	0.009 - 0.175	0.042	0.030	0.107	0.016 - 0.198	0.046	0.021	4.067	-1.148 - 9.282	2.661	0.127	-0.019	-0.134 - 0.096	0.059	0.747	\	\	\	\
time: group male with SWSD	0.101	0.018 -0.184	0.042	0.017	0.118	0.027 - 0.209	0.046	0.011	7.309	2.096 - 12.523	2.660	0.006	0.022	-0.093 - 0.137	0.059	0.710	\	\	\	\
time: Education	0.044	-0.085 - 0.174	0.066	0.504	0.055	-0.087 - 0.196	0.072	0.449	5.229	-2.915 - 13.374	4.155	0.209	0.073	-0.108 - 0.253	0.092	0.430	\	\	\	\
time: Occupation	0.031	-0.100 -0.162	0.067	0.640	0.036	-0.107 - 0.179	0.073	0.619	3.780	-4.443 - 12.002	4.195	0.368	0.153	-0.028 - 0.335	0.093	0.099	\	\	\	\
time: Alcohol	-0.009	-0.077 - 0.059	0.035	0.803	-0.013	-0.088 - 0.061	0.038	0.726	-1.177	-5.462 - 3.109	2.187	0.591	-0.061	-0.156 - 0.034	0.048	0.206	\	\	\	\
time: Smoking	0.031	-0.036 -0.098	0.034	0.366	0.037	-0.037 - 0.110	0.037	0.330	2.880	-1.360 -7.120	2.163	0.184	-0.050	-0.144 - 0.044	0.048	0.294	\	\	\	\
time: Exercise	0.017	-0.043 -0.077	0.031	0.580	0.014	-0.051 - 0.079	0.033	0.675	2.219	-1.545 - 5.982	1.920	0.248	0.087	0.004 - 0.170	0.043	0.041	\	\	\	\

Note. Predictors represent interaction terms between time and participant group indicators or covariates, including group membership (by sex and SWSD status) and lifestyle factors (education, occupation, alcohol use, smoking, and exercise). In the predictor labels, “with SWSD” and “without SWSD” indicate SWSD status.

For the models involving BCCs (WBC, NEU, LC, MC, and PLT) (Table 2, Figures 3A–E), the interaction between time and group was significant. In the NEU model, the slope of the SWSD group became steeper over time (time: group female with SWSD: *β* = 0.079, SE = 0.036, 95% CI [0.009, 0.149], *p* = 0.027; time: group male with SWSD: *β* = 0.076, SE = 0.036, 95% CI [0.007, 0.146], *p* = 0.032). Furthermore, in the PLT model, the slope for male with SWSD was significantly steeper (*β* = 4.752, SE = 2.011, 95% CI [0.811, 8.693], *p* = 0.018). To further explore group differences in slope, *post hoc* analyses with FDR correction were conducted. In the PLT model, no pairwise differences in slopes were observed (all FDR-adjusted *p* > 0.05). However, in the NEU model, the difference between the Female without SWSD and Female with SWSD was significant (FDR-adjusted *p* < 0.05), and the difference between the SWSD and non-SWSD groups was borderline significant (FDR-adjusted *p* = 0.050).

For the MLR, NLR, NMLR, SII, SIRI, and AISI models (as presented in Table 2; Figures 3F–H,K–M, 4F–H,K–M), the time × group interaction was significant for all inflammatory indices. Compared to the group without SWSD, the SWSD group exhibited a greater slope, indicating an upward trend in these inflammatory markers over time (all time: group female with SWSD and time: group male with SWSD: *p* < 0.05). *Post hoc* analyses with FDR correction indicated that the pairwise differences for the SWSD group were significant (FDR-adjusted *p* < 0.05).

In the PLR model (Table 2, Figures 3I, 4I), only the time × group interaction for males with SWSD was significant (*β* = 7.309, SE = 2.660, 95% CI [2.096, 12.523], *p* = 0.006). Post hoc analysis revealed significant differences only between the male with SWSD group and the male without SWSD group (FDR-adjusted p < 0.05), with no significant differences in other comparisons. Notably, no significant time × group interactions were observed in the MC, LC, WBC, dNLR (Figures 3J, 4J), or BMI (Figures 3N, 4N) models.

## 4 Discussion

In this three-year retrospective analysis, we used linear mixed-effects models to explore longitudinal changes in BCCs and related inflammatory indices among rotating shift workers, with a focus on the interaction between SWSD and sex. Across 14 inflammatory indices, we observed that participants with SWSD tended to show annual increases in NEU, MC and several composite inflammatory indices compared with those without SWSD, whereas sex-related differences were limited. PLR was the only marker showing a clear sex-related pattern, with an apparent upward trend in men with SWSD, although the overall interaction between PLR and sex was not significant.

A key strength of this work is the study setting and the way inflammation was characterized. We focused on newly employed rotating shift workers who continuously worked shifts over 3 years, and we relied on routinely collected annual BCCs rather than a single measurement. This design allows us to capture time-related changes that one-time assessments may miss, while keeping the approach practical and scalable because BCC-derived indices are inexpensive and widely available. Repeated measures may help support risk stratification and monitoring by flagging individuals with persistently unfavorable inflammatory profiles and by providing measurable targets for future schedule optimization or sleep-focused interventions.

Our observations are broadly aligned with previous reports that shift work disrupts circadian rhythms, alters clock gene expression, leads to sleep-related problems, triggers inflammatory responses, and increases levels of inflammatory markers (Dobrovinskaya et al., 2024; Pandey et al., 2025). Faraut et al. (2011) investigated the association between sleep restriction and immune cell dynamics and reported elevated NEU following sleep restriction (Li et al., 2022). They proposed that individuals with sleep disorders arising from prolonged shift work exhibit a gradual increase in WBC and NEU levels over time, potentially contributing to long-term adverse health outcomes, including increased mortality risk (Faraut et al., 2011). A review of WBC parameters in shift workers also documented higher total WBC, NEU and MC counts compared with day workers (Sooriyaarachchi et al., 2023), consistent with our observation that workers with sleep disorders related to shift work may experience low-grade systemic inflammation.

In our study, NEU levels increased earlier, notably in the second year, whereas MC changes became more evident in the third year. This temporal discrepancy may reflect the distinct dynamic roles of NEU and MC in SWSD-related inflammation. NEU are key components of the innate immune system and are typically among the first immune cells to be mobilized in response to physiological stress. Malfunctioning biological clocks may rapidly activate NEU, resulting in early increases in their levels. Our results are consistent with previous findings showing that NEU are particularly sensitive to sleep deprivation and may serve as an early indicator of inflammatory responses associated with sleep disorders (Lange and Born, 2011). In contrast, MC play a regulatory role and are involved in the chronic maintenance of inflammatory responses, with changes typically lagging behind those of NEU (Lee et al., 2009; Patel et al., 2021). In the present study, MC levels did not show a significant increase until the third year, potentially indicating progression from early acute inflammatory activation to a more prolonged, chronic, low-grade inflammatory state. MC are not only key mediators in chronic inflammation but can also differentiate into tissue macrophages or dendritic cells, contributing to tissue repair, immune tolerance, and activation of adaptive immunity (Patel et al., 2021). Therefore, the delayed increase in MC may reflect sustained immune activation, regulatory imbalance, or adaptive remodeling of the immune system in response to long-term SWSD.

The underlying mechanism responsible for the temporal difference between the early increase in NEU and the delayed change in MC is most likely also related to the influence of cortisol. Human circadian rhythms are regulated by an endogenous biological clock located in the suprachiasmatic nucleus (SCN) of the hypothalamus (Reppert and Weaver, 2002). Cortisol, a hormone controlled by circadian rhythms, is secreted through complex regulation of the HPA axis, which follows a precise circadian pattern (Panda et al., 2002). Night-shift work and irregular schedules can perturb the SCN and lead to chronic activation of the HPA axis (Andreadi et al., 2025), with elevated cortisol levels during the biological night. NEU are highly sensitive to cortisol, particularly during the early phases of stress (Obeagu, 2025). Elevated cortisol levels can promote the mobilization of NEU from the bone marrow into the circulation, inhibit apoptosis, and prolong its presence in the bloodstream by suppressing the expression of adhesion molecules (Klak et al., 2024; Obeagu, 2025). In contrast, MC exhibit complex regulatory responses to cortisol. Under acute stress, cortisol inhibits MC activation and migration. However, HPA axis hyperactivity over time can impair the negative feedback regulation of cortisol, leading to the differentiation of MC into pro-inflammatory subtypes (such as the M1 phenotype) and their accumulation in the peripheral blood (Yeager et al., 2016; Maciuszek et al., 2019). Notably, the development of a chronic inflammatory state is typically a slow and cumulative process, which likely requires prolonged circadian disruption and sustained exposure to elevated cortisol levels (Lawther et al., 2022; Xu et al., 2024). Thus, the earlier NEU changes and later MC changes we observed may reflect different phases of stress and inflammatory adaptation.

In the present study, multiple inflammatory indices (NLR, MLR, NMLR, SII, SIRI, and AISI) significantly increased over time in the SWSD group, and post-hoc comparisons suggested differences between participants with and without SWSD. These indices are composite scores calculated from BCCs and reflect the relative proportions and coordinated responses of different inflammatory indices subsets, including NEU, LC, MC, and PLT (Haryati et al., 2023; He et al., 2025). NLR and MLR are regarded as indicators of the balance between pro-inflammatory activity and lymphocyte-mediated immune regulation, whereas SII, SIRI, and AISI integrate multiple cellular components to provide a broader estimate of systemic inflammatory burden. The upward trends in these indices among rotating shift workers with sleep disorders may therefore be consistent with a pattern of chronic low-grade inflammation in this population. This interpretation aligns with previous reports suggesting that circadian rhythm disruption, impaired sleep quality, metabolic stress, and autonomic nervous system dysfunction in shift workers are associated with heightened inflammatory activity (Morris et al., 2017; Li X. et al., 2024; Yang et al., 2024). Experimental and clinical studies have also reported that chronic sleep disturbances are associated with activation of the HPA axis and enhanced sympathetic nervous system activity, together with sustained increases in proinflammatory mediators (Briançon-Marjollet et al., 2015; Mishra et al., 2020). These processes may be related to alterations in the composition and proportion of peripheral inflammatory cells and may help to contextualize the dynamic increases observed in inflammation-derived indices over time in our study.

Inflammation and SWSD are often discussed as a downstream consequence of circadian disruption, but the relationship may also operate in the opposite direction. Inflammatory signaling can reshape sleep regulation and circadian physiology, and ongoing sleep disruption may in turn sustain or amplify inflammatory tone, creating a reinforcing cycle (Piber et al., 2022). Mechanistic studies support this bidirectional view, implicating innate immune pathways and inflammasome-related signaling in linking sleep loss to neuroimmune activation and subsequent sleep–wake dysregulation (Amini et al., 2022). While our retrospective observational design does not allow conclusions about directionality, the time-related increases in BCC-derived indices observed in SWSD fit well within this feedback framework.

Among the BCCs and related inflammatory indices, PLR was the only marker that showed an apparent sex-related pattern, with a more pronounced upward trend in men with SWSD. This pattern may be related to sex hormone–associated differences in immune regulation (Ning et al., 2025). Variations in hormone concentrations, such as estrogen and testosterone, have been linked to differences in inflammatory responses. Testosterone levels in men can be suppressed during circadian disruption and have been associated with a more pro-inflammatory immune profile, whereas estrogen is generally considered to exert anti-inflammatory effects and may attenuate inflammatory activation in women. For instance, during COVID-19 infection, estrogen has been reported to inhibit excessive activation of innate immune cells while promoting CD4^+^ helper cell–mediated anti-inflammatory cytokine production (Pirhadi et al., 2020; Li et al., 2022). In addition, PLT activation has been associated with sleep disturbances. Emerging evidence suggests that, beyond their role in hemostasis, platelets participate in pathways related to chronic stress and inflammation through the release of pro-inflammatory mediators, regulation of endothelial function, and recruitment of immune cells (Karaganis and Zimmerman, 2024; Wang et al., 2024). In this context, the higher PLR observed in men with SWSD may reflect a sex-specific pattern of platelet and lymphocyte dynamics and may serve as a tentative indicator of a higher inflammatory burden in male shift workers (Mahdi, 2024). This observation highlights to the need for further studies to clarify sex differences in inflammatory profiles among individuals with SWSD.

This study had several limitations. We relied on existing records in a retrospective cross-sectional design, some categorical variables had to be coded more coarsely than ideal, and key exposures were derived from self-reported questionnaires; as a result, misclassification and reporting bias are possible (Tosoratto et al., 2024). The absence of a day-worker control group also narrows interpretation. In addition, unequal numbers of men and women constrained sex-stratified analyses and may have reduced the power to detect effect modification. Although blood draws were scheduled in the early morning, circadian phase was not standardized across individuals (e.g., time since the last night shift or sleep timing), leaving room for residual confounding due to diurnal variation in leukocyte subsets. Finally, we lacked information on hormonal contraceptive use, which could influence leukocyte profiles and derived inflammatory indices; pregnancy and breastfeeding were excluded to reduce major hormone-related confounding. These limitations indicate that the findings should be interpreted as preliminary and hypothesis-generating, and they support prospective studies with objective sleep and circadian assessments, sampling standardized to shift cycles, appropriate control groups, and linkage to clinical endpoints, as well as the development and validation of predictive models for SWSD-related risk stratification using readily available inflammatory markers.

## 5 Conclusion

This study shows that long-term rotating shift workers with SWSD exhibit increased levels of several inflammatory markers. The significant short-term elevation of NEU and sustained long-term changes in MC suggest that different immune cell types may play distinct dynamic roles in the chronic inflammatory response induced by circadian rhythm disruption. These findings indicate that tracking changes in inflammatory markers, particularly NEU and MC, may be useful for characterizing inflammatory profiles in rotating shift workers. Our results may help to generate hypotheses about potential time windows of SWSD-related inflammatory risk and to inform future studies on monitoring and intervention strategies for shift workers.

## Data Availability

The original contributions presented in the study are included in the article/supplementary material, further inquiries can be directed to the corresponding authors.
